# The Effect of Regimen Frequency Simplification on Provider Order Generation: A Quasi-Experimental Study in a Korean Hospital

**DOI:** 10.3390/ijerph18084086

**Published:** 2021-04-13

**Authors:** Jungwon Cho, Sangmi Shin, Young Mi Jeong, Eunsook Lee, Euni Lee

**Affiliations:** 1 Department of Pharmacy, Seoul National University Bundang Hospital, Seongnam-si 13620, Korea; xcully@snubh.org (J.C.); 30145@snubh.org (S.S.); escduck@snubh.org (E.L.); 2 College of Pharmacy & Research Institute of Pharmaceutical Sciences, Seoul National University, Seoul 08826, Korea

**Keywords:** dosing frequency, medication regimen simplification, health information systems, medical order entry systems

## Abstract

The multiplicity of dosing frequencies that are attached to medication orders poses a challenge to patients regarding adhering to their medication regimens and healthcare professionals in maximizing the efficiencies of health care service delivery. A multidisciplinary team project was performed to simplify medication regimens to improve the computerized physician order entry (CPOE) system to reduce the dosing frequencies for patients who were discharged from the hospital. A 36-month pre-test–post-test study was performed, including 12-month pre-intervention, 12-month intervention, and 12-month post-intervention periods. Two-pronged strategies, including regimen standardization and prioritization, were devised to evaluate the dosing frequencies and prescribing efficiency. The results showed that the standardized menu reduced the dosing frequencies from 4.3 ± 2.2 per day in the pre-intervention period to 3.5 ± 1.8 per day in the post-intervention period (*p* < 0.001). In addition, the proportion of patients taking medications five or more times per day decreased from 40.8% to 20.7% (*p* < 0.001). After prioritizing the CPOE dosing regimen, the number of pull-down options that were available reflected an improvement in the prescribing efficiency. Our findings indicate that concerted efforts in improving even a simple change on the CPOE screen via standardization and prioritization simplified the dosing frequencies for patients and improved the physicians’ prescribing process.

## 1. Introduction

The complexity of medication regimens performed during the care transitions of patients from hospitals to home- or community-based care settings poses a challenge to drug administration [1,2]. This complexity can be defined as the number of medications and the number of doses (dosing frequencies) prescribed to a patient [1]. Previous literature has highlighted that higher medication complexity decreased patient adherence [3,4,5], while the simplification of medication regimens not only improved medication adherence [6,7,8] but also reduced the burden of drug administration [7,8].

In Korea, the time of drug administration has been customarily anchored around mealtimes, such as “30 min after meals” or “right after meals.” Although mealtimes are important for medication guidance on special food–drug interactions, issuing routine patient instructions using mealtime-based drug labels has not been considered to be evidence-based practice [9]. Furthermore, hour-based dosing regimens (e.g., every eight hours) are prescribed when maximal drug effect is anticipated by considering the pharmacokinetic profile of the medication. In a system operating with various medication regimens, patients can experience difficulties in correctly taking their medications and the hardships may be even further amplified, especially when multiple medications are prescribed. If efforts are not made to consolidate these regimens, their complexity could pose a threat to patients and their caregivers, which could involve non-adherence and subsequent therapeutic failure [10,11,12].

Seoul National University Bundang Hospital (SNUBH), a tertiary care teaching hospital, has been supported by the computerized physician order entry (CPOE) system with a medication regimen menu, including the hour- and mealtime-based regimens, in the past. The end-users of the SNUBH system, i.e., clinicians, nurses, or pharmacists, have continuously raised the need for reducing the complexity of the medication regimens. Prescribers were hindered by a bulky and unstandardized menu for regimen selection when they entered a drug order. Additionally, pharmacists and nurses were burdened by complex and inefficient work processes while dispensing and administering the medications, respectively. The type and extent of the complexity could be diverse in other healthcare systems; as such, we believe that the problems of inefficiency could be a universal issue. Thus, SNUBH recruited a project team to address the problems with the complexity of medication regimens to specifically decrease dosing frequencies for patients by revamping the CPOE system in the health information system (HIS). While previous studies on changing medication regimens have largely focused on decreasing the number of medications using manual processes [13,14,15], we aimed to change our CPOE system by focusing on the regimen selection menu.

The objective of this study was to introduce a system approach to the problem-solving process and to evaluate whether changes in the CPOE system significantly improved the dosing frequencies for patients and the prescribing efficiency for physicians in the post-intervention period compared with those in the pre-intervention period.

## 2. Materials and Methods

### 2.1. Study Design and Period

**Figure 1:**
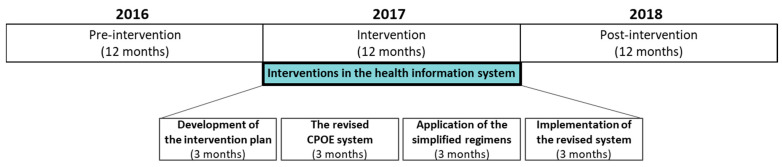
Study timeline, including the 12-month pre-intervention (January 2016–December 2016) and 12-month post-intervention (January 2018–December 2018) periods. The interventions (green box) were performed across 12 months (January 2017–December 2017). CPOE: computerized physician order entry.

This pre- and post-intervention study evaluated the effect of an initiative toward simplifying prescriber-focused medication, which was mainly facilitated by improving the CPOE system. The overall study lasted for 36 months (January 2016–December 2018), which was divided into three periods: 12-month pre-intervention (January 2016–December 2016), 12-month intervention (January 2017–December 2017), and 12-month post-intervention (January 2018–December 2018) (Figure 1).

### 2.2. Study Site

SNUBH is a 1335-bed tertiary care teaching hospital, where a fully digitized HIS has been used since the hospital was first opened in 2003. This HIS is homegrown and includes electronic medical records of patients’ demographic information, progress, medications, and laboratory results. Ordering medications electronically is possible with the CPOE system and a clinical decision support (CDS) system is applied to assist with prescribing medication orders. All information regarding the ordering of medications, including package labels, is managed and updated by the pharmacists through the HIS.

### 2.3. Study Process

**Figure 2:**
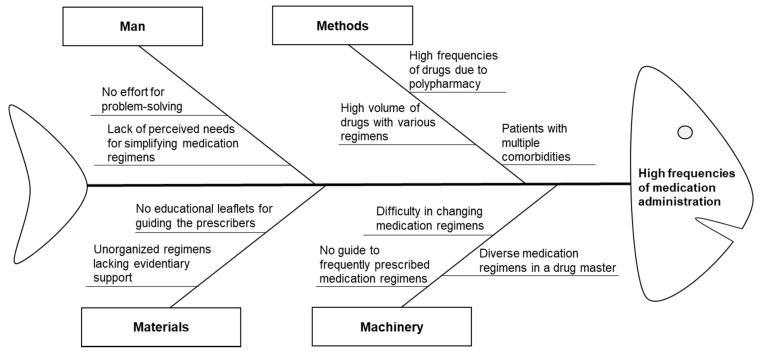
Fishbone diagram outlining barriers to reducing unnecessary dosing frequencies during medication administration.

A multidisciplinary team was recruited in December 2016, comprising two pharmacists, four attending physicians (one each from the departments of gastroenterology, nephrology, endocrinology, and neurology), two ward nurses, and a project manager in the department of medical informatics. The team developed an intervention plan and created a fishbone diagram using the 4 M’s approach to outline the potential barriers to simplifying the medication regimens in January 2017 (Figure 2). Based on their analysis, two CPOE enhancements for prescriber-focused medication simplification were proposed as interventions.

### 2.4. Interventions

The default medication regimens, including dose, dosing frequency, and route of administration, were provided as a prescriber-configurable pull-down option set to guide clinicians in selecting, deselecting, or switching to a different regimen. In the past, when clinicians had to change a regimen from the default regimen, they would face difficulties in finding appropriate regimens simply because there were too many regimen options (e.g., a total of 68 oral regimen options for once per day) and overlapping regimens (e.g., one hour before meals vs. two hours before meals; three times a day vs. every eight hours). Therefore, the enhanced regimens had to be simplified, prioritized, and reorganized to assist clinicians as they navigate the CPOE system to find the desired regimens. We revised the priority of dosage regimens in the CPOE system from an alphabetical order to a prescriber-friendly arrangement in terms of the prescribing frequency based on analyses of previous prescription data.

Consequently, two-pronged strategies, including standardization of the default regimens and prioritization of the prevalent regimens, were devised to enhance the CPOE system on medication regimens. We evaluated whether the changes in the system significantly improved the dosing frequencies and the proportion of patients taking medications with high frequencies in the post-intervention period compared with those in the pre-intervention period.

#### 2.4.1. Intervention 1: Standardization of the Default Regimens

The standardization of candidate regimens such as “30 min after meals” (1261 drugs) and “right after meals” (123 drugs) were unified into a single category: ”after meals.” In addition, default regimens for 65 drugs were revised within the range of the approved package labels provided by the Ministry of Food and Drug Safety in Korea. Table 1 presents the changes in regimens and the names of a few medications as examples. Drugs that had interactions with foods or should be administered in specific time intervals for maintaining an optimal blood concentration were excluded.

The above changes were initially reviewed by the team, and then attending physicians from six clinical departments (one each from the departments of endocrinology, gastroenterology, hematology/medical oncology, infectious diseases, nephrology, and neurology) verified the changes. The Pharmacy and Therapeutics Committee (P&T Committee), which included physicians, pharmacists, nurses, and administrators, was brought together to make the final decisions on the simplification process and complete the HIS build-up via a series of discussions among the P&T Committee members. The P&T Committee determined that no changes were to be made to the original regimens of anticancer agents and immunosuppressant drugs.

#### 2.4.2. Intervention 2: Prioritization of Prevalent Regimens

**Figure 3:**
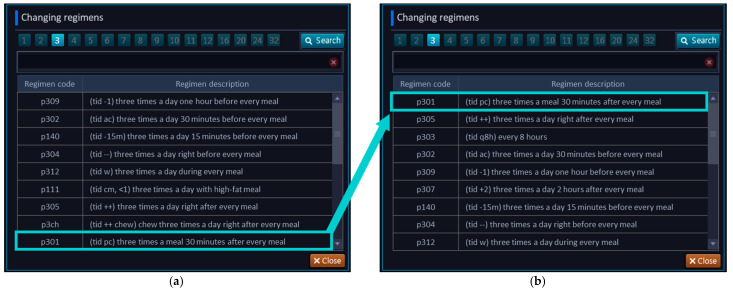
Rank changes of medication regimens in the CPOE system. Description of the CPOE system during the (**a**) pre-intervention and (**b**) post-intervention.

The medication regimens were prioritized and reorganized in the CPOE system to efficiently assist clinicians with placing a medication order based on the prescribing frequency. Among the previously prescribed 789 drug orders, 162 oral regimens were analyzed. Based on the analysis of the frequently used regimens, the display order in the CPOE system was prioritized in terms of the dosing frequency (Figure 3).

During the intervention period, changes in the CPOE system that were related to the medication regimens were disseminated to the system users, including clinicians, nurses, and pharmacists, with the support of leaders using posters on the bulletin boards of each floor in the hospital and demonstrating periodic advertisements at clinical meetings. Furthermore, the changes were shared with other external stakeholders, such as community pharmacists and the Regional Pharmacists Association, since the changed regimens were applicable to outpatient prescriptions written by the SNUBH prescribers.

### 2.5. Outcomes

The primary outcome was a reduction in the dosing frequency per day. The secondary outcome was a reduction in the proportion of patients taking medications with high frequencies (i.e., five or more times per day), as previously described in the literature [16], among the patients discharged during the study period. The study population excluded patients under 18 years or those discharged from special units, such as intensive care units, emergency rooms, and the delivery/maternity center. We also measured the efficiency of the regimen selection in the CPOE system by physicians. After the prioritization and reorganization in the CPOE system, the ranks of the pull-down options were calculated based on the prescribing frequency compared to that during the pre-intervention period. Consequently, these revised ranks were analyzed in the post-intervention period.

**Figure 4:**
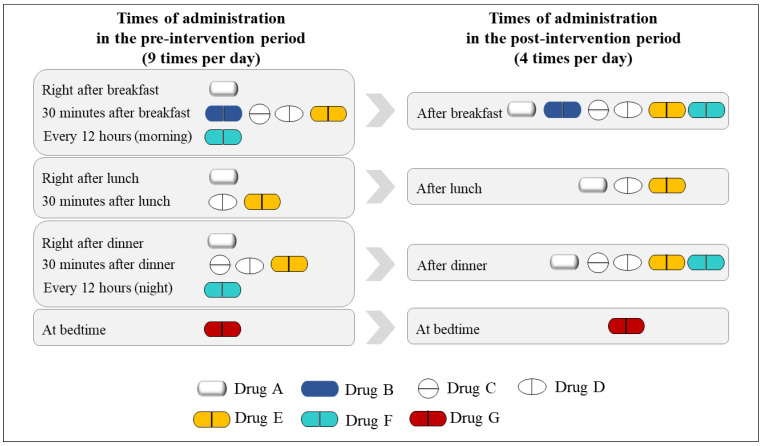
A case example of the improved dosage regimen for a patient.

The dosing frequency was calculated using the medication regimens described in each prescription by counting the different administration times separately. For example, if four medications were prescribed as follows: the first medication once a day after a meal, the second medication every 12 h, the third medication three times a day after each meal, and the fourth medication at bedtime, then the total dosing frequency per day would be six (three (after each meal) + two (every 12 h) + one (at bed time)). Figure 4 provides a case example of an improved dosage regimen in the pre- and post-intervention periods. A patient took the medicine nine times per day, as prescribed by the clinician in the pre-intervention period; however, the dosing frequency per day was decreased to four times per day in the post-intervention period. The improved dosage regimens were printed on the drug labels of each discharged medication.

### 2.6. Statistical Analysis

Our analysis was focused on the difference in the mean dosing frequency per day and the proportion of patients taking medications with high frequencies among the discharged patients. Descriptive statistics were used to summarize the patients’ demographics and the overall characteristics between the pre- and post-intervention groups. The characteristics of the two groups of patients who were discharged in the pre- or post-intervention periods were compared using Pearson’s chi-squared test and Student’s *t*-test for categorical and continuous variables, respectively. To analyze the differences between the pre- and post-intervention periods, Student’s *t*-test (for the mean dosing frequency per day) and Pearson’s chi-squared test (for the proportion of patients taking medications with high frequencies) were performed. All statistical analyses were performed in R version 4.0.2 2020 (The R Foundation for Statistical Computing, Vienna, Austria). A *p*-value of <0.05 was considered statistically significant for all statistical tests. 

### 2.7. Ethics Approval and Consent to Participate

This study was approved by the Institutional Review Board of SNUBH (B-1912-585-102) and a waiver for informed consent was obtained from the Institutional Review Board.

## 3. Results

### 3.1. Characteristics of the Study Population

During the study period, a total of 85,044 patients were discharged, of which, 40,716 patients were discharged in the pre-intervention period and 44,328 in the post-intervention period. The characteristics of the study population are presented in Table 2 by sex, age, length of stay, and department at discharge. Among the characteristics, age, length of stay, and department at discharge were statistically different between the two periods. 

### 3.2. Prioritization of Prevalent Regimens by Dosing Frequency

The result of the prioritization of prevalent regimens in the CPOE screen is presented in Table 3. The top three regimens in the post-intervention and the change in each regimen were compared in terms of the dosing frequency in the order in which the pull-down options were available. Meal-based regimens (e.g., 30 min after meals) and time-based regimens (e.g., every 8 h) were placed on the top of the CPOE screen. Consequently, the amount of scrolling required to select the prevalent medication regimens in the CPOE system was reduced in the post-intervention period. For example, the regimen “30 min after breakfast” used to be placed at rank 9 from the pre-intervention period but the new CPOE screen showed the regimen at rank 1 in the pull-down menu. 

### 3.3. Dosing Frequency per Day

The dosing frequency per day in the pre- and post-intervention periods were significantly different (Table 4). During the post-intervention period, the mean dosing frequency per day was significantly lower than that in the pre-intervention period (4.3 ± 2.2 vs. 3.5 ± 1.8 times, *p* < 0.001).

### 3.4. Proportion of Patients Taking Medications with High Frequencies

Table 4 shows the differences in the proportion of patients taking medications with high frequencies in the pre- and post-intervention periods. In the post-intervention period, the proportion of patients taking medications with high frequencies was significantly lower (9196 patients, 20.7%) compared with those in the pre-intervention period (16,619 patients, 40.8%) (*p* < 0.001). 

## 4. Discussion

We evaluated the impact of the homegrown HIS changes on medication simplification to improve the CPOE system and to reduce the dosing frequencies for patients. Simplification of the medication regimen refers to the process of consolidating the dosing frequency or standardizing the routes of administration [17,18]. As the number of older adults taking multiple medications is increasing [19], reducing the complexity of medication regimens can be considered the main strategy to improve medication adherence and subsequent clinical outcomes. 

One of the key highlights of our study was the approach adopted to facilitate a system change, with the help of multidisciplinary healthcare professionals in a hospital. Many published studies have reported their successful pharmacy interventions using a chart review [14,15], consultation teams [20,21], or simple notifications [22]. Interventions directed at physicians are essential for improving the medication use process; however, these can be time-consuming and often impractical [19]. In addition, interventions for healthcare professionals could involve repetitive feedbacks and were often dependent on the contents of the intervention in terms of how these professionals received such feedbacks [23,24]. Unlike other studies conducted, interventions on medication simplification by changing direct services delivered by healthcare professionals [25,26,27,28], we believe that the significance of our study was in its approach to provide a system change. As Schneider et al. described, over 95% of health institutions in the United States use the CPOE system [29]; therefore, our study demonstrating our collaborative approach toward changing the HIS can be instrumental in enhancing this system’s feasibility, serve as a potentially adaptable method to enhance the CPOE system in other healthcare settings, and prove to be a methodological tool for real-world-based intervention studies.

With the two-pronged HIS interventions for the standardization of default regimens and the prioritization of prevalent regimens, our study findings documented that coordinated changes in the CPOE system could improve the experiences of many stakeholders in the medication use process, including patients, caregivers, and prescribing physicians. The dosing frequency per day in the post-intervention period was significantly improved. Consequently, the proportion of patients taking medications with high frequencies was significantly lower as well. We believed that some characteristics should be compared between the pre- and post-interventions because changes in the prescription patterns that are specific for sex or age groups could confound the changes in the patterns of simplified medication regimens. For patients and their caregivers, the intervention simplified the dosing frequencies during their care transition. For physicians, their prescription-ordering process became more efficient by prioritizing and reorganizing the regimen selection menu while avoiding excess choices [30], and potentially reduced the possibilities for making new types of errors associated with the CPOE system, such as typing and pull-down option errors [31].

This study has some limitations. We performed a pre- and post-intervention study, which is a quasi-experimental research design and has the inherent limitations of non-randomized, uncontrolled study designs. Second, we confirmed the impact of the intervention on simplifying the medication regimens on the transition of care, specifically for the efficiency of prescribing medications. However, we could not provide details on the clinical outcomes at the level of each patient. Although our study did not include individual clinical outcomes to demonstrate the impact of simplifying the medication regimen, we believe that our findings could serve as a basis for future research using HIS. Third, our study could not evaluate the potential benefits of the CPOE system changes for other stakeholders in the medication use process, such as system builders, pharmacists, or nurses. Fourth, we only evaluated the short-term effects produced by the interventions across one year. Future studies should examine the long-term effects to evaluate the sustainability of the HIS intervention. Lastly, our study has limited generalizability as we only evaluated the effect of HIS on simplifying the medication regimen at one institution. Further studies are needed to confirm whether other medical institutions also achieve a similar effect. However, since mealtime-based regimens are still customarily used in other hospitals in Korea, our findings are meaningful, as such regimens need to be revised to standardize the evidence-based regimens. Consequently, we believe that exploring these systematic changes can provide opportunities for accomplishing global standards for patient care.

## 5. Conclusions

Our findings highlight the fact that prescriber-focused medication simplification significantly reduced the dosing frequency per day and the proportion of patients taking medications with high frequencies among discharged patients. Key interventions included two enhanced HIS components: the standardization of default regimens and the prioritization of prevalent regimens. We believe that our results, which were derived from a comprehensively planned project with HIS interventions and combined with promotions, demonstrate effective and sustainable changes, which can be adapted to other healthcare institutions that employ CPOE systems.

## Figures and Tables

**Table 1:** Simplified medication regimens and examples.

Changes	*n*	Examples
Right after meal → after meal	123	Azathioprine
30 min after meal → after meal	1261	Amlodipine besylate
One tablet every eight hours → one tablet three times a day	10	Acetaminophen
One tablet every 12 h → one tablet twice a day	48	Cefuroxime axetil
One tablet every 24 h → one tablet once a day	7	Moxifloxacin

**Table 2:** Characteristics of the study population.

Characteristics	Pre-Intervention ^1^ *n* = 40,716 (%)	Post-Intervention ^2^ *n* = 44,328 (%)	*p*-Value
Sex			
Female	20,480 (50.3)	22,541 (50.9)	0.11 ^a^
Male	20,236 (49.7)	21,787 (49.1)	
Age, mean ± SD (years)	57.9 ± 16.9	58.6 ± 16.8	<0.001 ^b^
18–65 years	25,302 (62.1)	27,403 (61.8)	0.33 ^a^
Over 65 years	15,414 (37.9)	16,925 (38.2)	
Length of stay, mean ± SD (days)	10.8 ± 30.6	9.9 ± 25.8	<0.001 ^b^
Department at discharge			<0.001 ^a^
Internal medicine	13,565 (33.3)	14,767 (33.3)	
Surgery	9503 (23.3)	10,644 (24.0)	
Obstetrics and gynecology	3865 (9.5)	4725 (10.7)	
Orthopedics	3466 (8.5)	3529 (8.0)	
Urology	3061 (7.5)	2928 (6.6)	
Neurology	1576 (3.9)	1607 (3.6)	
Neuropsychiatry	935 (2.3)	865 (2.0)	
Pediatrics	72 (0.2)	120 (0.3)	
Others	4673 (11.5)	5143 (11.6)	

^1^ 1 January 2016–31 December 2016. ^2^ 1 January 2018–31 December 2018. SD, standard deviation. ^a^ Pearson’s chi-squared test, ^b^ Student’s *t*-test.

**Table 3:** Prioritization of the top three prevalent regimens by dosing frequency on the CPOE screen.

Dosing Frequency	Regimens	Pull-Down Options’ Order		
		Post	Pre	Rank Change
QD	30 min after breakfast	1	9	8↑
	Right after breakfast	2	8	6↑
	Before sleep	3	36	33↑
BID	30 min after breakfast and dinner	1	8	7↑
	Right after breakfast and dinner	2	6	4↑
	30 min after breakfast and before sleep	3	14	11↑
TID	30 min after each meal	1	9	8↑
	Right after each meal	2	7	5↑
	Every 8 h	3	14	11↑
QID	30 min after each meal and before sleep	1	6	5↑
	Right after each meal and before sleep	2	5	3↑
	Every 6 h	3	10	7↑

QD, quaque die (once a day); BID, bis in die (two times a day); TID, ter in die (three times a day); QID, quater in die (four times a day).

**Table 4:** Medication regimen outcomes in the pre- and post-intervention periods.

Outcomes	Pre-Intervention ^1^ *n* = 40,716 (%)	Post-Intervention ^2^ *n* = 44,328 (%)	*p*-Value
Mean dosing frequency per day, count (SD)	4.3 (2.2)	3.5 (1.8)	<0.001
Proportion of patients taking medications with high frequencies, *n* (%)	16,619 (40.8)	9196 (20.7)	<0.001

^1^ 1 January 2016–31 December 2016. ^2^ 1 January 2018–31 December 2018.

## Data Availability

Data sharing is not applicable to this article.
